# Weight gain prevention among black women in the rural community health center setting: The Shape Program

**DOI:** 10.1186/1471-2458-12-305

**Published:** 2012-06-15

**Authors:** Perry Foley, Erica Levine, Sandy Askew, Elaine Puleo, Jessica Whiteley, Bryan Batch, Daniel Heil, Daniel Dix, Veronica Lett, Michele Lanpher, Jade Miller, Karen Emmons, Gary Bennett

**Affiliations:** 1 Duke Obesity Prevention Program, Duke Global Health Institute, 2812 Erwin Road, Suite 403 Box 90392, Durham, NC, 27705, USA; 2 School of Public Health and Health Sciences, University of Massachusetts Amherst, 425 Arnold House 715 North Pleasant Street, Amherst, MA, 01003-9304, USA; 3 College of Nursing and Health Sciences, University of Massachusetts Boston, 100 Morrissey Boulevard, Boston, MA, 02125, USA; 4 Division of Endocrinology, Metabolism and Nutrition, Duke University Medical Center, 200 Trent Drive, Duke South Orange Zone DUMC, Box 3031, Durham, NC, 27710, USA; 5 Department of Health & Human Development, Montana State University, H&PE Complex, Hoseaus Room 121, Bozeman, MT, 59717, USA; 6 Dana-Farber Cancer Institute, 450 Brookline Avenue, LW601, Boston, MA, 02215, USA

**Keywords:** Obesity, Weight, eHealth, Women’s health, Minority health, Primary care, Prevention

## Abstract

**Background:**

Nearly 60% of black women are obese. Despite their increased risk of obesity and associated chronic diseases, black women have been underrepresented in clinical trials of weight loss interventions, particularly those conducted in the primary care setting. Further, existing obesity treatments are less effective for this population. The promotion of weight maintenance can be achieved at lower treatment intensity than can weight loss and holds promise in reducing obesity-associated chronic disease risk. Weight gain prevention may also be more consistent with the obesity-related sociocultural perspectives of black women than are traditional weight loss approaches.

**Methods/Design:**

We conducted an 18-month randomized controlled trial (the Shape Program) of a weight gain prevention intervention for overweight black female patients in the primary care setting. Participants include 194 premenopausal black women aged 25 to 44 years with a BMI of 25–34.9 kg/m^2^. Participants were randomized either to usual care or to a 12-month intervention that consisted of: tailored obesogenic behavior change goals, self-monitoring via interactive voice response phone calls, tailored skills training materials, 12 counseling calls with a registered dietitian and a 12-month YMCA membership.

Participants are followed over 18 months, with study visits at baseline, 6-, 12- and 18-months. Anthropometric data, blood pressure, fasting lipids, fasting glucose, and self-administered surveys are collected at each visit. Accelerometer data is collected at baseline and 12-months.

At baseline, participants were an average of 35.4 years old with a mean body mass index of 30.2 kg/m^2^. Participants were mostly employed and low-income. Almost half of the sample reported a diagnosis of hypertension or prehypertension and 12% reported a diagnosis of diabetes or prediabetes. Almost one-third of participants smoked and over 20% scored above the clinical threshold for depression.

**Discussion:**

The Shape Program utilizes an innovative intervention approach to lower the risk of obesity and obesity-associated chronic disease among black women in the primary care setting. The intervention was informed by behavior change theory and aims to prevent weight gain using inexpensive mobile technologies and existing health center resources. Baseline characteristics reflect a socioeconomically disadvantaged, high-risk population sample in need of evidence-based treatment strategies.

**Trial registration:**

The trial is registered with clinicaltrials.gov NCT00938535.

## Background

The epidemic of obesity in the U.S. shows no signs of abating – presently, almost 70% of the adult U.S. population is either overweight or obese[1]. Black women are disproportionately affected by the condition. Between 1976 and 2008, obesity among black women increased more than 60%[2,3]. Nearly 60% of black women are obese, a rate that is twice that of non-Hispanic white women[1]. Socioeconomic status and obesity are less strongly associated in black women than in other groups. Nevertheless, socioeconomic factors strongly pattern exposure to obesogenic environmental factors [4,5], the adoption of obesogenic risk behaviors[6], the limited availability of weight management resources[7,8], and the efficacy of obesity treatment strategies in the primary care setting[9].

Despite their vastly increased risk of obesity and associated chronic disease[10,11], racial/ethnic minority and socioeconomically disadvantaged populations have been underrepresented in clinical trials of weight loss interventions[11]. This is problematic because promoting weight loss among black women is a long-standing and vexing clinical challenge[12,13]. Evidence-based obesity treatments are consistently less effective and absolute weight losses are generally smaller among black women, compared to other populations[10,11,14]. There is growing recognition that alternative clinical treatment strategies are necessary to contend with the challenge of obesity[14-17]. While it is undeniable that weight loss is the optimal treatment strategy for many obese individuals, weight gain prevention may have considerable clinical utility among overweight and some obese black women.

Weight gain prevention holds promise in reducing risk associated with cardiovascular diseases (CVD), type 2 diabetes, some cancers[18] and perhaps premature mortality[19]. Weight gain prevention may have particular benefits for blacks, who exhibit disproportionately greater rates of adulthood weight gain[20,21] and extreme obesity [2], both of which increase obesity-associated chronic disease risk[22-24]. Relative to whites, black women have weaker associations of adiposity with cardiovascular risk factors[25-28] and mortality from cardiovascular disease[29,30] and all causes. Thus, promoting *weight stability* within the overweight (BMI = 25-29.9 kg/m^2^) and lower levels of the Class 1 obesity ranges (BMI = 30-34.9 kg/m^2^) might be an appropriate chronic disease risk reduction strategy in black women, especially prior to menopause, when weight gains are particularly pronounced[31,32].

Additionally, we suspect that weight gain prevention strategies may be more consistent with the sociocultural experiences of black women, compared to traditional weight loss approaches. While some opposing data exist, most studies have shown that black women are more tolerant of heavier body weights, as compared to white women[33]. Blacks have a greater social acceptance of overweight, less body weight dissatisfaction, and higher body weight ideals than do whites[12,33-40]. A number of studies have shown that overweight blacks are less likely to perceive themselves to be overweight, compared to whites and Hispanics[41-43]. Perceived body image and attractiveness are not as strongly linked with weight in black women, compared to white women. Moreover, a majority of blacks do not consider overweight to be unhealthy[43]. Given that black women’s views about attractiveness and health are not closely associated with their weight status, weight loss messages[44-46] – which emphasize the importance of thinness – may have limited effectiveness among obese women. Intervention messages that emphasize weight gain prevention or enhancement of one’s current shape may have greater sociocultural relevance, thus enhancing participant receptivity[47,48].

Given the challenges associated with promoting weight loss among black women, particularly in the primary care setting, alternative treatment strategies are necessary. Weight gain prevention among overweight and Class 1 obese individuals is one such approach, one that requires relatively low treatment intensity and might be more consistent with the sociocultural experiences of black women. We suspect that its lower intensity and greater consistency with sociocultural norms may heighten participant responsiveness, improve intervention engagement, and enhance intervention outcomes among black women.

## Methods/Design

We conduct the Shape Program (Shape), an 18-month randomized controlled trial of a weight gain prevention intervention for overweight and Class 1 obese (BMI: 25–34.9 kg/m^2^) black female patients in the primary care setting. The primary outcome is weight maintenance over 12 months; secondary outcomes are change in obesity risk behaviors and obesity-related biomarkers, as well as maintenance of outcomes through 18-months. The primary hypothesis is that baseline BMI levels will be maintained in participants randomized to the intervention, while BMI levels will increase in those assigned to usual care.

All study procedures and protocols were approved by the Duke University Institutional Review Board and the Piedmont Health Board of Advisors.

## Setting

Shape is conducted in six CHCs operated by Piedmont Health, a private, non-profit community health system that operates six health centers in a seven-county service area in central North Carolina. Each Piedmont Health center offers primary care services, with additional site-specific services (e.g., laboratory, dentistry, pharmacy) that address local needs. Registered dietitians based at each health center provide WIC counseling, diabetes education, and medical nutrition therapy. Piedmont Health has a patient population of nearly 40,000 with over 123,900 medical/dental visits in 2010. Patients are predominately racial/ethnic minority (77%), 98% are <200% of the federal poverty level, and most are either uninsured, underinsured, or hold public insurance (59% uninsured, 31% Medicaid/Medicare).

## Participants

Participants include 194 premenopausal black women, aged 25 to 44 years, with a BMI of 25-34.9 kg/m^2^. Additional inclusion criteria are: at least one visit to a Piedmont Health center in the prior 24 months, North Carolina residency, and the ability to read and write in English. Exclusion criteria include: current pregnancy, being ≤ 12 months postpartum, a history of myocardial infarction or stroke in the prior two years, and profound cognitive, developmental or psychiatric disorders.

## Participant screening and recruitment

Recruitment of participants occurred between September 2009 and February 2011. Piedmont Health staff used electronic medical record (EMR) data to generate lists of potentially eligible patients from each health center. Study staff abstracted patients’ heights and weights from paper medical charts to assess BMI eligibility (25–34.9 kg/m^2^).

Potential participants were sent invitation letters (signed by the respective health center medical director and the study principal investigator) and study brochures via postal mail. Patients could opt out of the study by calling the toll-free number provided in the recruitment letter. No patients opted out of the recruitment process. After one week, study staff called potentially eligible patients to invite participation, perform an initial eligibility assessment, and schedule a screening evaluation visit.

## Randomization

Randomization occurred at the baseline visit, using a computer-based algorithm that was triggered after participants completed the baseline questionnaire battery. The randomization algorithm allocated participants equally (1:1) across treatment arms. We assigned participants to one of two research assistants and participants randomized to the intervention arm were randomly assigned to one of two interventionists. The intervention design precluded blinding either patients or interventionists to treatment assignment.

## Sample size

The study is designed to detect a difference of 1.03 kg/m^2^ in BMI at the 0.05 alpha level and 80% statistical power using a two-tailed test for differences. We increased the target sample size to account for examination of effect modifiers and mediators.

## Treatment arms

### Usual care

Usual care participants received the current standard of care offered by their primary care providers. In addition, usual care participants received semi-annual newsletters from our study team over the 12-month project period. These newsletters covered topics (e.g., finances, the environment) that were relevant to women in the target age group but did not relate to weight, nutrition, or physical activity.

### Weight maintenance intervention

#### Theoretical framework

Social Cognitive Theory (SCT) [49,50] informed the intervention’s design. From SCT, self-efficacy was selected as the primary psychosocial mediator. There is strong and consistent evidence that self-efficacy is positively associated with weight loss intentions, initiation, and maintenance[51-53]. The intervention was designed to target each of the four factors that Bandura identified as influencing self-efficacy: [54] mastery experiences, social modeling, social persuasion, and somatic and emotional reactions. Social Cognitive Theory also indicates that behavior change can be facilitated through a number of self-regulatory processes that were built into the intervention, including self-monitoring[55,56], goal setting[53,57], and social support[58]. The intervention was designed to support these self-regulatory processes, which should further increase self-efficacy.

#### Intervention design

The intervention contained five components (Table 1): 1) obesogenic behavior change goals; 2) self-monitoring via interactive voice response (IVR) phone calls; 3) tailored skills training materials; 4) 12 interpersonal counseling calls; and 5) a 12-month YMCA membership.

**Table 1:** Intervention design

**Component**	**Type of contact**	**Frequency of contacts (over 12-month period)**
Self-monitoring with tailored feedback	Printed tracking logs IVR calls	Daily 1/week
Tailored skills training	Printed materials	22/year
Interpersonal counseling	Coaching calls from Registered Dietician	1/month
Physical activity	YMCA membership	12-month membership

#### Behavior change goals

The intervention utilized the interactive obesity treatment approach (iOTA), which creates an energy deficit sufficient to produce weight change through the modification of routine obesogenic lifestyle behaviors[59,60]. Participants were assigned 3 behavior change goals from the iOTA library using an algorithm that considers a participant’s need for change, self-efficacy, readiness, and the goal’s intended caloric deficit. The iOTA goal library contains over 21 obesogenic behavior change goals (e.g., five or more fruits and vegetables/day, no fast food, no sugar sweetened beverages, walking 7,000 steps/day) that were selected based on their: 1) empirical support; 2) population relevance; 3) ease of self-monitoring; and 4) concreteness. Participants were assigned new goals at months two and four to maintain motivation and facilitate goal mastery. At the beginning of each new goal assignment (every two months), participants received printed personalized feedback reports that detailed results of the previous goal assignments as well as provided tailored prescriptions for new assignments.

### Behavior change strategies

#### Self-monitoring

We recommended that participants self-monitor their iOTA behavior change goals daily using paper tracking logs. Participants were given pedometers to facilitate daily monitoring of physical activity. Participants relayed the self-monitoring data recorded on their tracking logs to the study team during weekly interactive voice response (IVR) calls. Interactive voice response calls allow one to interact with a computer system using a telephone by typing on the keypad or via speech. Participants received weekly IVR calls throughout the 12-month intervention. Self-monitoring data collected via IVR were visible to coaches to inform counseling activities during monthly coaching calls.

After self-monitoring data was collected, tailored feedback was immediately provided through IVR. Feedback messages described trends in participant progress, reinforced successes, and/or offered motivational strategies. Short skills training tips were also provided.

Shape utilized telephonic technologies for several reasons. First, IVR helps to overcome the literacy/numeracy barriers associated with detailed paper records. These systems have high reach[61,62], as mobile phone penetration is very high in the target population. In contrast to web-based approaches, telephony is easily accessible, low-cost, quickly used, and requires no expert knowledge. Finally, IVR is inexpensive to develop, simple to tailor and immediately scalable.

#### Skills training materials

A major point of innovation in the Shape design is that, unlike traditional weight loss strategies, our approach suggests that participants maintain their current weight (and shape). This approach inherently embraces long-held social norms and aesthetic values. The intervention’s focus resulted from qualitative pilot work designed to assess the acceptability of print materials and to test narrative messages.

Shape print materials were tailored at several levels. Participants received skills training content that corresponded to their behavior change goals. At baseline visits, participants were provided with a set of tailored intervention materials to be utilized over the first two months of the intervention. For example, individualized “Shape Tracking Logs” included tailored narratives based on each participant’s unique set of goals. Additional materials were sent via postal mail every two months. We sent cycle-specific materials every two months in order to keep participants focused on the goals of current assignment and to heighten feelings of novelty and connection with the program. Participants also received quarterly newsletters with additional skills training information (e.g., appropriate portion sizes, food shopping tips, healthy recipes).

#### Telephone counseling calls

Each month, 20-minute counseling calls were delivered by Piedmont Health registered dietitians (“coaches”) trained in motivational interviewing principles[63]. The coach calls were designed to enhance self-efficacy by guiding participants through identification of barriers to behavior change and resulting ambivalence towards change efforts. They also provided skills training and helped participants utilize goal-setting as a problem solving strategy.

Coaches used a web application that presented each session’s call script, allowed for note taking, and provided access to participant self-monitoring data. The system recorded calls and automatically stored process data (e.g., date/time, call disposition, duration). Prior to the monthly counseling calls, coaches reviewed the participant self-monitored IVR goal tracking data that was held on the centralized data management system. The coaches used this data to guide discussions of participant progress towards assigned goals and to discuss readiness for behavior change and barriers to change. Data from the coaching calls were stored on the secure study server with all other study data for monitoring and data collection purposes.

Shape coaches participated in a 2-day training session at baseline and received biannual refresher trainings. Shape staff monitored IVR data for completeness and reviewed 5% of coaching calls for adherence to protocol. Weekly supervision with the intervention coordinator ensured appropriate delivery of the coaching component of the intervention.

#### YMCA

Participants in the target population have limited options for safe and affordable physical activity. We addressed this barrier and promoted participant motivation for physical activity by providing intervention participants with 12-month memberships to local YMCAs.

### Data collection

At the screening visit, research assistants oriented participants to the study, gathered informed consent, and collected anthropometric data to confirm BMI eligibility. The anthropometric and blood data collection activities were conducted at baseline and again at study follow-up visits at 6, 12, and 18 months.

#### Anthropometric data

Participants changed into hospital gowns and their body heights were measured to the nearest 0.1 cm using a calibrated wall-mounted stadiometer (Seca 214) [64] and body weights were measured to the nearest 0.1 kg using a portable electronic scale (Seca Model 876) [64]. Waist circumference was measured to the nearest 0.1 cm using a vinyl, retractable tape measure (AccuFitness MyoTape) where circumference was measured horizontally from the highest point of the iliac crest at minimal respiration. The Omron HEM 907XL, a microprocessor controlled, noninvasive device that automatically measures systolic pressure, diastolic pressure, and pulse rate for adults, was used to measure blood pressure three times at 1-minute intervals after five minutes of quiet sitting. Participants were advised not to smoke or to consume any caffeine within 30 minutes of their study visits.

#### Cardiometabolic biomarkers

Participants were instructed to fast for at least eight hours prior to their study visits. Each participant had a fasting glucose and lipid panel analyzed using fingerstick blood specimens collected in 40μl capillary tubes (Cholestech LDX; Cholestech Corporation, Hayward, CA, USA).

#### Physical activity

All participants (Intervention and Usual Care) wore accelerometers (Actical; Philips Respironics, Inc., Bend, OR USA) on their non-dominant wrists[65] to provide estimates of free-living physical activity before baseline and after 12-month visits. Participants were instructed to wear the monitors continuously until their return visits approximately 14 days later. Upon its return, the activity monitor was removed from the wrist and data was downloaded to a computer and visually screened for compliance and collection errors. Complete files were defined as those in which the monitor had been worn for ≥10 days (i.e., complete compliance).

Activity monitor files were first transformed from 15-sec to 1-min epochs and then “smoothed” using a protocol validated for wrist monitoring of physical activity in overweight and obese adults[66]. Next, the resulting data were transformed into units of activity energy expenditure (AEE; kcals/kg/min) using a 2R calibration algorithm[67] and then summarized into outcome variables that included time (T; mins/week) and activity energy expenditure (AEE; kcals/week) engaged in light (T_L_ and AEE_L_, respectively) and moderate-vigorous (T_MV_ and AEE_MV_, respectively) activity intensities. The cut point used to distinguish light from moderate intensity activities was 0.0385 kcals/kg/min, which is the same value defined previously for overweight and obese adults[68]. Finally, both T_MV_ and AEE_MV_ variables were summarized in activity bouts of 1 and 10 minutes. These data-screening and processing procedures were consistent with those recently recommended for use with accelerometry-based physical activity data[69].

#### Survey data

Participants complete self-report surveys at baseline and 6-, 12-, and 18-month follow-up. Surveys are administered via computer using an online survey tool (http://www.surveygizmo.com). Demographic variables collected at baseline include age, race/ethnicity, marital status, occupational status, educational attainment, income, and co-morbidities.

We used validated measures to assess a range of relevant constructs, including:

##### Body image

The 14-item Figure Rating Scale (FRS) is designed to assess current and past body size as well as attractiveness of body figure drawings [70].

##### Quality of life

The 5-item EuroQol instrument (EQ-5D) assesses mobility, self-care, usual activities, pain/discomfort, and anxiety/depression. The EuroQol visual analog scale (EQ-VAS) is similar to a health thermometer and is designed to assess self-rated health quality of life[71,72].

##### Medical history

Twelve items measure general health and previous diagnosis and perceived risk of diabetes and high blood pressure.

##### Physical activity

Four items are used to assess current stage of change in relation to physical activity. A 6-item scale from the Behavioral Risk Factor Surveillance System (BRFSS) is designed to assess the amount and frequency of moderate and vigorous activity[73,74].

##### Tobacco use

Three items assess current smoking behaviors and previous quit attempts. This measure is derived from the National Health Interview Survey (NHIS) [75].

##### Sleep behavior

The Medical Outcomes Study (MOS) Sleep is a 12-item measure designed to assess minutes to fall asleep, total hours of sleep each night, and difficulties with sleep. The questionnaire assesses six dimensions: sleep disturbance, sleep adequacy, daytime somnolence, snoring, short of breath, and quantity of sleep[76].

##### Self-efficacy for exercise

Self-efficacy for exercise is measured using five items that assess confidence in ability to exercise when tired, in a bad mood, don’t have time, on vacation, or when it is raining/snowing[77].

##### Genetic causal beliefs

An 8-item scale is designed to assess perceptions of risk for obesity, diabetes, and heart disease (i.e., genetic or behavioral lifestyle habits) [78,79].

##### Dietary restraint

The Three Factor Eating Questionnaire is designed to assess different dimensions of eating behavior. For the current study, we used the 18-item revised version (TFEQ-R18) to measure three domains: Cognitive Restraint (6 items), Uncontrolled Eating (9 items), and Emotional Eating (3 items) [80,81].

##### Social support

A 19-item subscale from the Medical Outcomes Study (MOS-SSS) assesses availability of social support. Four subscales are used: emotional/informational, tangible, affectionate, and positive social interaction[82].

##### Perceived weight

A 12-item scale is designed to assess perceptions of past, current, and future weight, self-perceived weight class (i.e., underweight, average weight, overweight), and body satisfaction.

##### Negative life events

A 16-item questionnaire measures frequency of stressful life events[83].

##### Depression

The 8-item Patient Health Questionnaire (PHQ-8) is designed to assess the presence of depressive symptoms[84].

##### Health literacy

A 3-item questionnaire is used to screen for limited health literacy[85].

##### Neighborhood environment

The physical activity environment questions include items adapted from the Neighborhood Environment Walkability Scale (NEWS) on perceptions of the built environment, land use mix, and community support for physical activity [86].

##### Food security

An adaptation of the USDA Household Food Security Scale (6 items) is designed to assess household food security (money for food, food affordability, skipped meals) [87-89].

##### Racial identity

The 8-item Centrality subscale of the Multidimensional Inventory of Black Identity (MIBI) is used to measure African-American or black racial identity[90].

##### Absenteeism and presenteeism

The 11 items from the World Health Organization Health and Work Performance Questionnaire (HPQ)-Short Form assess number of hours worked, expected hours of work, missed work, total hours of work over the past 4 weeks, and perceived job performance[91,92].

### Data analysis

Shape will be evaluated using the RE-AIM planning and evaluation framework[93] (Table 2). The RE-AIM framework addresses five issues related to both internal and external validity by comprehensively evaluating the success of interventions on issues key for translation from research to practice and dissemination: 1) **R**each and representativeness of individuals who participate; 2) **E**ffectiveness/Efficacy of the intervention on the primary outcomes at the individual level; 3) **A**doption at the organizational/CHC level; 4) **I**mplementation measured at the CHC provider/staff level; and 5) **M**aintenance at both the individual participant and provider level.

**Table 2:** RE-AIM Measures

**Domain**	**Description**[94]	**Measure**	**Data source(s)**
Reach	Degree to which target population is reached by study activities	1. % Eligible population contacted 2. % Who respond to contact 3. % Who participate/are excluded 4. Representativeness of study sample to target population	**1-4.** Study database **1**–**4.** PHS EHR
Efficacy	Improvement in study outcomes	1. Change in weight and secondary outcome measures	**1.** In-person measurement
Adoption	Potential organizational uptake	1. Patient intervention satisfaction	**1.** Survey (12 mo)
Implementation[95]	Degree to which intervention is implemented as intended	1. Interventionist adherence to counseling protocol 2. Participant adherence to intervention	**1.** Study database **1.** Process measures including: supervisor review of coach call recordings, of # attempted and completed coach calls, of timing and accuracy of mailed materials **1.** Patient survey (baseline, 6, 12 mo) **2.** # attempted and completed coach and IVR calls **2.** # participant YMCA visits
Maintenance	Can program outcomes be sustained over time?	1. Weight change at 18 months	**1.** Patient survey (18 mo) **1.** PHS EHR
Cost	How much does intervention cost?	1. Cost of staff time (coach and research staff) devoted to conducting intervention activities 2. Cost of intervention delivery: print materials, technology costs, YMCA memberships	**1.** Study database **1.** Staff time diaries **2.** Financial statements

### Baseline characteristics

A total of 194 black female patients were randomized to treatment arms. Five participants became ineligible after randomization due to pregnancy or diagnosis of cancer. At baseline (Table 3), participants were an average of 35.4 (SD = 5.5) years old with an average BMI of 30.2 (SD = 2.6) kg/m^2^. Participants were mostly employed (70.4%) and low-income – 73.5% had an annual household income ≤ $29,999 and one-third lived beneath the federal poverty threshold. Participants were supporting an average of 3.2 (SD = 1.3) persons with their household income. Most participants (72.5%) did not live with partners in the household. One-third of the sample had a high school diploma, GED or less and only 7% had completed college.

**Table 3:** Baseline characteristics of the Shape Program analytic sample (n = 189)

**Variable**	**N (percent)**
Race/ethnicity	
Black or African American	189 (100.0)
More than 1 race	11 (5.8)
Education	
Less than high school graduate	19 (10.0)
High school graduate or GED	45 (23.8)
Some college or vocational/trade school	83 (43.9)
Associate degree	26 (13.8)
College graduate or post graduate degree	13 (6.9)
Unknown	3 (1.6)
Annual household income	
Under $10,000 - $29,999	139 (73.5)
$30,000 - $39,999	24 (12.7)
Over $40,000	24 (12.7)
Unknown	2 (1.1)
People supported by this income: mean (SD)	3.2 (1.3)
Living under U.S. Census poverty threshold	62 (33.3)
Marital status	
Married	48 (25.4)
Widowed, divorced, separated	44 (23.3)
Never married	78 (41.3)
Not married or living with partner	15 (7.9)
Unknown	4 (2.1)
Current employment	
Yes	133 (70.4)
No	53 (28.0)
Unknown	3 (1.6)
Current smoker	58 (30.7)
Self-reported history of:	
Diabetes or prediabetes	23 (12.2)
Hypertension or prehypertension	87 (46.0)
Meeting U.S Federal guidelines for physical activity using accelerometers	49 (31.6)
Depression score: mean (SD)	6.2 (4.9)
Depression score ≥ 10	38 (20.1)
	**Mean (SD)**
Age (yrs)	35.4 (5.5)
Weight (kg)	81.2 (8.8)
BMI (kg/m^2^)	30.2 (2.6)
Waist circumference (cm)	97.8 (8.2)
Blood pressure: systolic (mmHg)	123.2 (14.8)
Blood pressure: diastolic (mmHg)	80.6 (11.0)
Lipids	
Triglycerides (mg/dL)	102.2 (47.5)
LDL (mg/dL)	107.4 (34.2)
HDL (mg/dL)	53.8 (16.1)
Total cholesterol (mg/dL)	179.2 (37.3)
Fasting glucose (mg/dL)	105.1 (44.3)

Accelerometers placed at screening visits were returned an average of 15.1 days after initial placement. Complete baseline accelerometer data was collected for 87.6% of the participants (n = 170). Nearly a third (31.6%) of participants met the federal guideline of ≥ 150 minutes of 10-minute bouts of physical activity each week. Participants averaged 166.9 (SD = 265.5) minutes a week of moderate physical activity in 10-minute bouts and no (0.0) minutes a week of vigorous activity in 10-minute bouts.

Almost half (46.0%) of the sample reported a diagnosis of hypertension or prehypertension and 12% reported a diagnosis of diabetes or prediabetes. Almost one-third of participants smoked and over one-fifth scored above the PHQ clinical threshold for depression. Mean blood pressure measurements were: SBP = 123.2 (SD = 14.8)/DBP = 80.6 (SD = 11.0). Mean lipid levels were optimal or close to optimal.

## Discussion

Despite the recent plateau of the obesity epidemic in the U.S. adult population [1], black women still have dramatically higher obesity risk compared to other groups. Given evidence suggesting that overweight (and Class 1 obesity) is less health damaging for black women compared to other racial/ethnic groups, maintaining weight status among black women in the BMI ≤ 35 kg/m^2^ range may hold promise as an alternative obesity treatment strategy. Strategies are needed that can prevent weight gain for women in the BMI ≤ 35 kg/m^2^ range, those who might benefit most from weight gain prevention approaches. Fortunately, weight gain prevention can be accomplished at lower treatment intensity than can weight loss [96]. A major advantage for this population is that weight gain prevention builds upon existing weight-related sociocultural norms among black women instead of challenging them. In stark contrast to the focus of weight loss interventions, Shape’s content focused on “maintaining shapes” and “showing off curves” in order to validate sociocultural norms about body image and to reinforce self-affirming weight maintenance messages.

Furthermore, Shape was designed for primary care practice – a particularly meaningful setting for addressing the obesity epidemic and one in which relatively few weight-related trials are conducted. More specifically, the intervention was developed for implementation in community health centers, critical primary care delivery systems for our nation’s most medically vulnerable populations. Indeed, the baseline characteristics of the Shape sample reflect a group that is at extremely high risk for obesity and obesity-associated chronic disease. The sample was composed of largely rural, black women who are unmarried, supporting several family members, and struggling to make ends meet. About half of the sample was diagnosed with hypertension or prehypertension. Participants demonstrated elevated levels of moderate physical activity (that was likely due primarily to occupational pursuits) and no vigorous activity, which suggests little intentional exercise. Many women (over 20%) in our sample were above the clinical threshold for depression. Together, this group is one at extremely high disease risk and, yet, one for which we have few evidence-based intervention approaches.

Although Shape is a novel approach executed in an understudied population, we are encouraged by our initial success with recruitment and retention. To optimize participant recruitment and retention, the study team employed several strategies designed to accommodate the busy lives of study participants, most of whom work, are socioeconomically disadvantaged, and are solely responsible for children in the household. Study visits were offered in a number of locations convenient to participants, including in the Piedmont Health centers, at all times of day on weekdays and weekends. Participants who notified the study team about transportation difficulties were offered home visits and taxi vouchers for evaluation visits.

The research team worked closely and collaboratively with Piedmont Health partners on Shape design and implementation issues. The research team designed recruitment and data collection activities to be minimally burdensome on health center practices, while harnessing the strengths of the Piedmont Health system. Financial mechanisms were negotiated that adequately reimbursed Piedmont Health for its involvement and for the staff time of its registered dietitians, who functioned as study coaches. These types of strategies are crucial in ensuring that CHC-based research benefits patients without overburdening already-strained clinical operations.

In conclusion, black women are disproportionally affected by the epidemic of obesity and, consequently, the risk of comorbidities associated with being obese. In previous trials, low-income black women have not been as well represented as other groups; when included, their weight loss outcomes have been suboptimal. Shape was designed to take an innovative approach to managing obesity and its health consequences among black women by integrating behavior change theory, building upon an understanding of sociocultural norms and utilizing health information technologies. In these ways, Shape was designed to be responsive to calls for interventions that have dissemination potential in real world practice settings. Such considerations are particularly pressing for socioeconomically disadvantaged populations given their dramatically increased risk of obesity and obesity-associated chronic diseases and the limited availability of obesity treatment options.

## Abbreviation

CVD = Cardiovascular disease; CHC = Community health center; EMR = Electronic medical record; SCT = Social cognitive theory; iOTA = Interactive obesity treatment approach; IVR = Interactive voice response; AEE = Activity energy expenditure; T = Time; FRS = Figure rating scale; BRFSS = Behavioral risk factor surveillance system; NHIS = National health interview survey; MOS = Medical outcomes study; PHQ = Patient health questionnaire; MIBI = Multidimensional inventory of black identity.

## Competing interests

The authors declare that they have no competing interests.

## Authors’ contributions

PF managed study design and execution and drafted the manuscript for publication. EL and JW coordinated intervention design. BB consulted on data safety and execution of the study. DH consulted on the accelerometer data collection protocols and analyzed accelerometer data. EP and SA participated in study design and conducted statistical analysis. DD, VL, ML and JM conducted primary data collection and participated in study design. KE participated in study conceptualization and design. GB conceived of the study, acquired study funding, participated in study design and coordination and drafted the manuscript for publication. All authors read and approved the final manuscript.

## Authors’ information

DD is now with Quintiles Transnational in Durham, NC and VL is now with the School of Social Work at the University of North Carolina, Chapel Hill, NC.

## Pre-publication history

The pre-publication history for this paper can be accessed here:

http://www.biomedcentral.com/1471-2458/12/305/prepub
